# Are We fulfilling the Hippocratic Oath?

**DOI:** 10.5005/jp-journals-10071-23144

**Published:** 2019-04

**Authors:** Atul P Kulkarni

**Affiliations:** Division of Critical Care Medicine, Tata Memorial Hospital, Homi Bhabha National Institute, Mumbai, Maharashtra, India

## Abstract

**How to cite this article:** Kulkarni AP. Are We fulfilling the Hippocratic Oath? Indian J Crit Care Med 2019;23(4):163.

**Table:** 

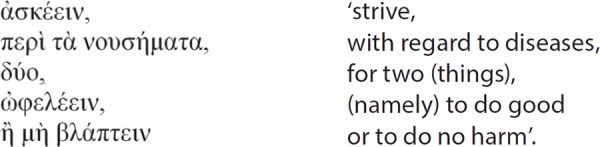

In our attempt to do good for our patients, are we actually harming our patients? Most critically ill patients are on multiple drugs. A combination of drugs might be harming the patients without us knowing about it.

In this issue of the journal, Wagh and colleagues unmask a hidden monster lurking in every intensive care unit (ICU), i.e., potential drug–drug interactions (pDDIs).^1^ They found that that each ICU patient had at least eight drugs in each prescription (8.8 + 3.35). No wonder then, that they found 76.25% incidence of pDDIs. Out of 1,171 pDDIs, 715 (61%) were major. A large number of interactions (44.24%) occurred in the elderly patients (>60 years). The mechanism of pDDIs was described as pharmacodynamic in nature.

We prescribe so many pharmacotherapeutic agents for our patients without realizing that these may lead to major pDDIs of which a large proportion may be major (D), or contraindicated (X) interactions.

Why should the intensivist worry about these pDDIs? pDDIs may be a reason for admission in a large number of patients, cause increased ICU length of stay (LOS) and may lead to adverse drug reactions.^2^

Baniasadi and colleagues found that the common classes of drugs involved in pDDIs were antimicrobials, central nervous and cardiovascular medications, the drugs acting on the gastrointestinal system and lastly the hormones and their synthetic analogs. The commonest mechanisms involved were metabolism, absorption, and less common mechanism was additive effects, and rarely excretion and antagonism.^3^

What is the solution to this omnipresent threat? If we adopt computerized drug prescription, monitor the drug therapy, and have clinical pharmacist on the multidisciplinary team rounds, we can reduce the incidence of DDIs.^4^ Malfará and colleagues studied the effect of pharmacotherapist intervention in a pediatric ICU.^5^ The pharmacotherapist intervened (at least once) in 42% of patients (median two interventions per patient). Nearly 30% of these interventions were related to allergies, drug interactions, and therapeutic monitoring. The intervention also prevented under or overdosing of medications. All of this led to reduced pDDIs and cost saving to the tune of 15,118.73 Brazilian Real (US$ 4828.00). Other researchers have advised adoption of these strategies and also some other way to tackle this potential menace. Monitoring the patient for clinical evidence of drug toxicity apart from therapeutic effects of drugs, monitoring the laboratory parameters, and electrocardiogram (ECG) and other monitoring. Using clinical decision support systems linked to laboratory data and prescription data have also been suggested.^6^ After daily evaluation for pDDIs, using Micromedex and Lexi-Interact interaction databases in 400 patients, Somithberger and colleagues found 1,150 potential interactions, which could have resulted in 287.5 pDDIs per 100 patient-days. A large number of these pDDIs (5–9%) were either major (D) or contraindicated (X). They advocated active surveillance to prevent potential harm to the patients.^7^ Alas, in India, expecting to have all of these facilities in the same unit is delusional.

Further research in this neglected aspect in the critical care is the obvious need of the hour. The Indian Society of Critical Care of Medicine can formulate the recommendation to identify, monitor, and treat this problem. This elephant in the room needs to be confronted and brought to heal, to improve safety of our critically ill patients.
